# Safety and Efficacy of Contemporary Drug-Eluting Stents in Patients With ST-Segment Elevation Myocardial Infarction and a High Ischemic Risk

**DOI:** 10.3389/fcvm.2022.880351

**Published:** 2022-05-23

**Authors:** Oh-Hyun Lee, Yongcheol Kim, Nak-Hoon Son, Deok-Kyu Cho, Jung-Sun Kim, Byeong-Keuk Kim, Donghoon Choi, Myeong-Ki Hong, Myung Ho Jeong, Yangsoo Jang

**Affiliations:** ^1^Yonsei University College of Medicine Cardiovascular Center, Yongin Severance Hospital, Yongin-si, South Korea; ^2^Data Science Team (Biostatistician), Center for Digital Health, Yongin Severance Hospital, Yongin-si, South Korea; ^3^Department of Statistics, Keimyung University, Daegu, South Korea; ^4^Severance Cardiovascular Hospital, Yonsei University Health System, Seoul, South Korea; ^5^Chonnam National University Hospital and Medical School, Gwangju, South Korea; ^6^Department of Cardiology, CHA Bundang Medical Center, CHA University School of Medicine, Seongnam-si, South Korea

**Keywords:** ST-segment elevation myocardial infarction, drug-eluting stent, percutaneous coronary intervention (PCI), target lesion failure, high risk factor

## Abstract

**Background:**

In patients with ST-elevation myocardial infarction (STEMI) with a high risk of ischemic events, the safety and efficacy of drug-eluting stent (DES) are unclear.

**Methods:**

Based on the nationwide, multicenter, prospective registry, we selected 1,592 patients who underwent primary percutaneous coronary intervention (PCI) with everolimus-(EES) and zotarolimus-eluting stent (ZES) for STEMI with a high risk of an ischemic event. The occurrence of target lesion failure (TLF) for 3 years, defined as the composite of cardiac death, target vessel myocardial infarction (TV-MI), and ischemia-driven target lesion revascularization (ID-TLR), was evaluated.

**Results:**

The prevalence of high ischemic risk features was observed in 43.4% (2,744/6,325) of overall patients with STEMI. Among them, a total of 1,078 and 514 patients were treated with EES and ZES, respectively. At 3 years, the risk of TLF was not significantly different between the two groups (*p* = 0.93). In addition, the incidence of cardiac death, TV-MI, ID-TLR, and definite/probable stent thrombosis (ST) were also not different between the two groups. Moreover, elderly patients (age > 75 years) and PCI for the left main disease were identified as independent predictors of TLF.

**Conclusion:**

Implantation of EES or ZES provided comparable clinical outcomes in STEMI patients and high ischemic risks.

## Graphical Abstract

Visual abstract. DES, drug-eluting stent; STEMI, ST-segment elevation myocardial infarction; DM, diabetes mellitus; CKD, chronic kidney disease; uLMs, unprotected left main stem; PCI, percutaneous coronary intervention; TLF, target lesion failure; MACE, major adverse cardiac event; EES, everolimus-eluting stent; ZES, zotarolimus-eluting stent. 
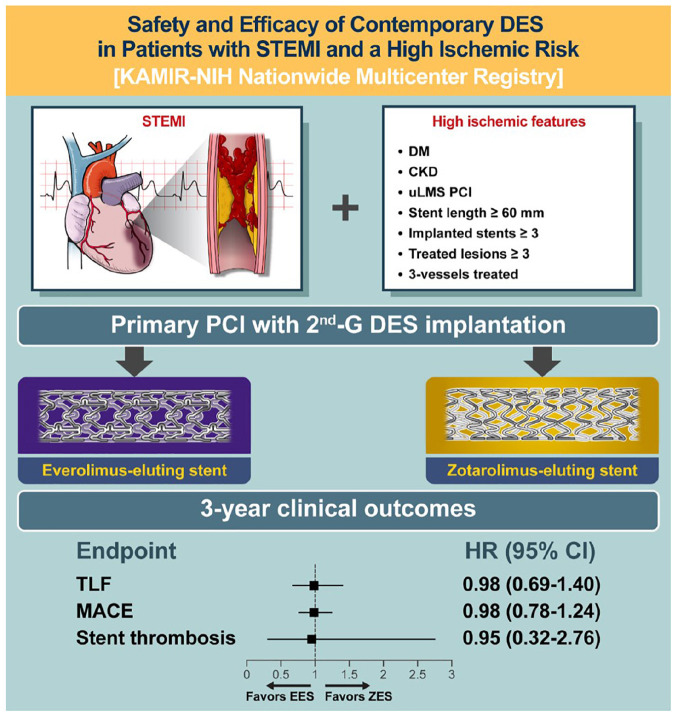

## Introduction

Acute myocardial infarction (AMI) is one of the leading causes of mortality and morbidity worldwide. Although the clinical outcomes have gradually improved owing to the widespread adoption of urgent or primary percutaneous coronary intervention (PCI) and recent pharmacological and technical developments, the short-term mortality and long-term mortality are still high, especially in patients with ST-segment elevation myocardial infarction (STEMI) (1, 2). Furthermore, the interventional techniques and device advances, such as coronary stents, have led to an increase in percutaneous coronary intervention (PCI) in patients with clinically and anatomically substantial ischemic risks. Recently, considering the clinical and anatomical complexity association with future cardiovascular events (3), high ischemic risk concepts of such clinical characteristics and coronary artery lesion-related or procedural complexity have been introduced (4, 5).

In the STEMI setting, the current guidelines recommend primary PCI with second-generation drug-eluting stent (DES) implantation as the default strategy because it demonstrated better efficacy than a bare-metal stent (BMS) or first-generation DES, in particular with respect to the lower ischemic events, such as stent thrombosis (ST) and myocardial infarction (5, 6). However, there is a paucity of data regarding clinical outcomes of second-generation DESs in STEMI patients with a high ischemic risk. Thus, the present study is aimed to evaluate the safety and efficacy of contemporary DESs in STEMI patients with a high risk of ischemic events.

## Materials and Methods

### Study Design and Subjects

The study population in the current study was based on the Korea Acute Myocardial Infarction Registry-National Institute of Health (KAMIR-NIH) registry, which is a nationwide, multicenter, prospective registry of patients with AMI in the Republic of Korea without application of any exclusion criteria. The 20 nationwide tertiary cardiovascular centers eligible for primary PCI and onsite cardiac surgery were recruited. The detailed study protocol has been previously published (7). All data were assessed by independent clinical research coordinators using a web-based case report form in the Internet-Based Clinic Research and Trial management system (iCReaT). It has been supported by a grant from the Korea Centers for Disease Control and Prevention, Ministry of Health and Welfare, the Republic of Korea since November 2011 (iCreaT study no. C110016, cris.nih.go.kr: KCT-0000863). Each participating center’s ethics committee approved the study protocol (CNUH-2011-172). This study complied with the Declaration of Helsinki provisions. All patients provided written informed consent to participate in the registry.

**FIGURE 1:**
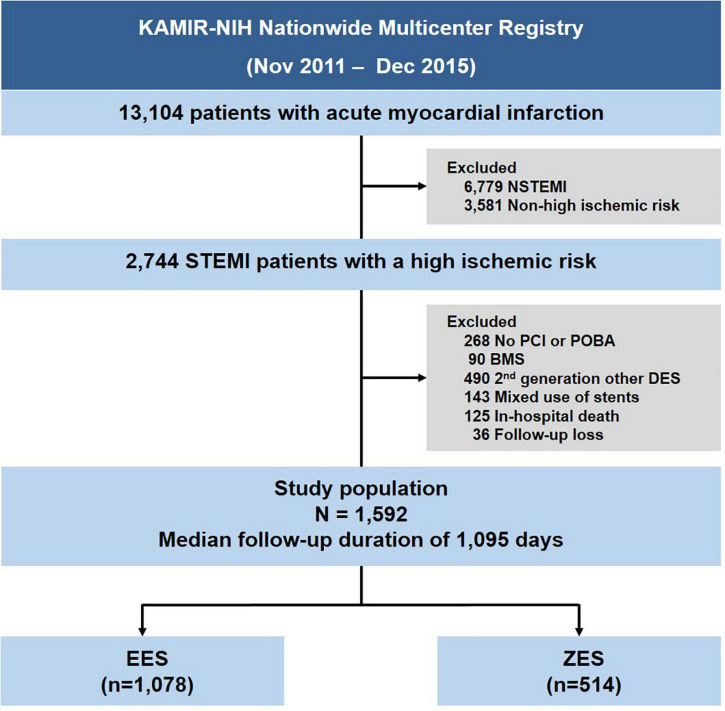
Study flow chart. STEMI, ST-elevation myocardial infarction; NSTEMI, non-ST-segment elevation myocardial infarction; PCI, percutaneous coronary intervention; POBA, plain old balloon angioplasty; BMS, bare-metal stent; DES, drug-eluting stent; EES, everolimus-eluting stent; ZES, zotarolimus-eluting stent.

The study population’s selection is shown in Figure 1. Among the 13,104 consecutive patients with AMI enrolled between November 2011 and December 2015, we selected STEMI patients with a high ischemic risk, which was defined according to previous studies as the presence of the following: diabetes mellitus (DM) or chronic kidney disease (CKD) history (estimated glomerular filtration rate ≤ 60 ml/min/1.73 m^2^), PCI for unprotected left main stem (uLMS) disease, stent length ≥ 60 mm, implanted stents ≥ 3, treated lesion ≥ 3, or 3-vessels treated (5, 8, 9). The exclusion criteria were no PCI or PCI without stenting, patients treated with BMS, first-or other second-generation DESs, mixed use of stent types, in-hospital death, and patients lost to follow-up. We defined lost to follow-up as when the patient was discharged alive but never visited the outpatient department. As a result, 1,592 patients were selected for this analysis; these patients were then divided into those who underwent PCI with EES [Xience prime, Xience Expedition, Xience Alpine (Abbot Vascular, Santa Clara, MA, United States), Promus Elements or Promus Premier (Boston Scientific, Natick, MA, United States)], and ZES [Resolute Integrity and Resolute Onyx (Medtronic, Santa Rosa, CA, United States)] (Supplementary Table 1).

### Study Procedures

Patients diagnosed with AMI were treated according to contemporary guidelines (10, 11). All patients received antiplatelet agents, such as aspirin (300 mg) and a P2Y_12_ inhibitor (clopidogrel 300–600 mg, ticagrelor 180 mg, or prasugrel 60 mg), before the procedure. After PCI, daily aspirin (100 mg) and P2Y_12_ inhibitors (clopidogrel 75 mg once, ticagrelor 90 mg twice, or prasugrel 10 mg once daily) were prescribed as a maintenance dose. All procedures were performed with standard interventional techniques. The treatment strategies, such as vascular access, glycoprotein IIb/IIIa inhibitors use, intravascular imaging modalities, or thrombosuction, were determined at the operator’s discretion.

### Definitions and Outcomes

The primary end point was target lesion failure (TLF), defined as the composite of cardiac death, target vessel myocardial infarction (TV-MI), and ischemia-driven target lesion revascularization (ID-TLR) at 3 years. TV-MI was defined as MI attributable to a target vessel. Target lesion revascularization (TLR) was considered ischemia-driven if any revascularization, such as the target lesion’s PCI or bypass surgery, was undertaken in the presence of ≥50% angiographic diameter stenosis with ischemic symptoms or a positive functional study or a ≥70% angiographic diameter stenosis with or without documented ischemia. The secondary end point included TLF’s individual components, definite/probable ST as defined by the Academic Research Consortium definitions (12), and major adverse cardiovascular event (MACE), which comprised a composite of death from any cause, MI, and revascularization.

### Statistical Analysis

Continuous variables were expressed as mean ± standard deviation (SD) or median (interquartile range) based on data normality and were compared using an independent sample *t*-test or Mann–Whitney U test, as appropriate. Categorical variables were presented as numbers and percentiles and compared using the chi-square test. Cumulative incidence of events at 3 years was calculated based on Kaplan–Meier censoring estimates, and clinical outcome comparisons between the two groups were performed with the log-rank test.

Sensitivity analyses were performed to adjust for confounding factors. First, a multivariable Cox regression model was used. Covariates that were statistically significant on univariate analysis (*p* < 0.10) were included: DM, clopidogrel usage, multiple treated vessels (≥2), total stent number, and imaging-guided PCI. Second, to reduce the selection bias and other potential confounding factors, we performed an analysis using the logistic regression model with inverse probability of treatment weighting (IPTW). The adjusted covariates in the IPTW analysis included DM, clopidogrel usage, multiple treated vessels, total stent number, and imaging-guided PCI (Supplementary Table 2).

We established a multivariable Cox proportional hazards model to identify independent predictors of 3-year TLF and MACE. The primary end point comparison according to the various exploratory subgroups was followed. In all analyses, the participating centers were included as random effects. A two-sided value of *p* < 0.05 was considered statistically significant. Statistical analyses were performed using SPSS statistical software (SPSS version 23.0 for Windows, IBM Corp., Armonk, NY, United States).

## Results

### Baseline Characteristics

In patients with STEMI, clinical and lesion- and procedure-related high ischemic risk features incidence was 43.3% (2,744/6,325). Among STEMI patients with a high ischemic risk, primary PCI with EES implantation was performed in 1,078 patients and ZES implantation in 514, respectively. The mean age of the patients was 65.0 ± 11.8 years (range: 33–93 years), and 1,164 (74.5%) were men. The baseline clinical and lesion- and procedure-related characteristics of the two groups are summarized in Tables 1, 2. Compared to the ZES group, the EES group had higher proportions of individuals with multiple treated vessels (≥2) and treated with multiple stents (≥2). More stents were implanted and less usage of intravascular imaging was observed during PCI in the EES group than in the ZES group. In IPTW analysis, baseline, angiographic, and procedural covariates were similar between EES and ZES groups (Supplementary Tables 3, 4).

**TABLE 1:** Baseline characteristics.

	Crude population		
	
	EES (*n* = 1,078)	ZES (*n* = 514)	*p*-value
Age, y	64.9 ± 11.7	65.2 ± 12.1	0.66
Male gender, n (%)	806 (74.8)	379 (73.7)	0.66
Body mass index,†kg/m^2^	24.0 ± 3.2	24.2 ± 3.1	0.28
Hypertension, n (%)	623 (57.8)	307 (59.7)	0.46
Diabetes mellitus, n (%)	559 (55.6)	313 (60.9)	0.04
Dyslipidemia, n (%)	131 (12.2)	72 (14.0)	0.30
Current smoker, n (%)	412 (38.2)	194 (37.7)	0.86
Prior myocardial infarction, n (%)	74 (6.9)	35 (6.8)	0.97
Prior cerebrovascular accident, n (%)	75 (7.0)	38 (7.4)	0.75
Killip class ≥ 3, n (%)	198 (18.4)	89 (17.3)	0.61
LVEF ≤ 40%, n (%)	51 (24.2)	17 (17.5)	0.19
Cardiogenic shock	117 (10.9)	59 (11.5)	0.71
**Laboratory findings**			
Peak troponin I, pg/mL	79.7 ± 104.6	72.7 ± 109.8	0.27
LDL-cholesterol, mg/dL	109.3 ± 38.7	110.0 ± 38.1	0.75
Creatinine, mg/dL	1.2 ± 0.8	1.2 ± 0.9	0.94
Hemoglobin, g/dL	13.8 ± 2.0	13.9 ± 2.2	0.71
Platelet count, 10^3^/μL	231.2 ± 64.5	237.3 ± 69.7	0.10
**Discharge medication, n (%)**			
Aspirin	1069 (99.2)	513 (99.8)	0.18
P2Y_12_ inhibitor			
Clopidogrel	748 (69.4)	383 (74.5)	0.04
Prasugrel	100 (9.3)	39 (7.6)	0.26
Ticagrelor	225 (29.1)	88 (26.7)	0.43
ACEi or ARB	845 (78.4)	397 (77.2)	0.61
Beta-blocker	949 (88.0)	448 (87.2)	0.62
Calcium channel blocker	42 (3.9)	24 (4.7)	0.47
Statin	1014 (94.1)	476 (92.6)	0.27

*ZES, zotarolimus-eluting stent; EES, everolimus-eluting stent; LVEF, left ventricular ejection fraction; LDL, low-density lipoprotein; ACEi, angiotensin-converting enzyme inhibitor; ARB, angiotensin receptor blocker.*

*Data are presented as mean ± standard deviation or number (%).

*†The body mass index is the weight in kilograms divided by the square of the height in meters.*

**TABLE 2:** Angiographic and procedural characteristics.

	Crude population		
	
	EES (*n* = 1,078)	ZES (*n* = 514)	*p*-value
Trans-radial approach, n (%)	225 (20.9)	113 (22.0)	0.61
Target vessel, n (%)			0.23
LM	31 (2.9)	15 (2.9)	
LAD	527 (48.9)	236 (45.9)	
LCX	81 (7.5)	29 (5.6)	
RCA	439 (40.7)	234 (45.5)	
CAD extent, n (%)			0.26
CAD 1VD	430 (39.9)	221 (43.0)	
CAD 2VD	397 (36.8)	191 (37.2)	
CAD 3VD	251 (23.3)	102 (19.8)	
LM involvement, n (%)	69 (6.4)	24 (4.7)	0.17
Lesion type B2 or C, n (%)	972 (90.2)	450 (87.5)	0.11
Multiple treated vessels (≥2), n (%)	329 (30.5)	127 (24.7)	0.02
Multiple stents (≥2), n (%)	465 (43.1)	192 (37.4)	0.03
Mean stent diameter, mm	3.13 ± 0.42	3.16 ± 0.40	0.22
Total stent number	1.7 ± 0.9	1.5 ± 0.8	<0.01
Total stent length, mm	34.6 ± 16.8	33.4 ± 16.4	0.18
Imaging-guided PCI, n (%)	209 (19.4)	122 (23.7)	0.046
Thrombus aspiration, n (%)	351 (32.7)	179 (35.4)	0.29
Glycoprotein IIb/IIIa inhibitor use, n (%)	234 (21.7)	97 (18.9)	0.19

*ZES, zotarolimus-eluting stent; EES, everolimus-eluting stent; LM, left main artery, LAD, left anterior descending artery; LCX, left circumflex artery; RCA, right coronary artery; CAD, coronary artery disease; LM, left main; PCI, percutaneous coronary intervention.*

**FIGURE 2:**
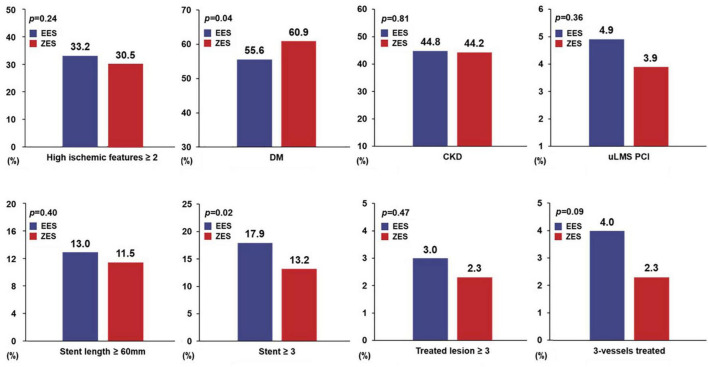
Prevalence of high ischemic risk features. EES, everolimus-eluting stent; ZES, zotarolimus-eluting stent; DM, diabetes mellitus; CKD, chronic kidney disease; uLMS PCI, unprotected left main stem percutaneous coronary intervention.

Figure 2 presents the trend in high ischemic risk features in both groups. The prevalence of patients with high ischemic features ≥ 2 was similar between the two groups (EES vs. ZES, 33.8 vs. 30.8%, *p* = 0.24), but more patients with DM were observed in the ZES group than in the EES group (EES vs. ZES, 55.6 vs. 60.9%, *p* = 0.04). On the other hand, the proportion of patients treated with ≥3-stent implantation was significantly higher in the EES group (EES vs. ZES, 17.9 vs. 13.2%, *p* = 0.02). The prevalence of other factors was similar between the two groups.

### Comparison of 3-Year Clinical Outcomes According to Drug-Eluting Stent Types

**FIGURE 3:**
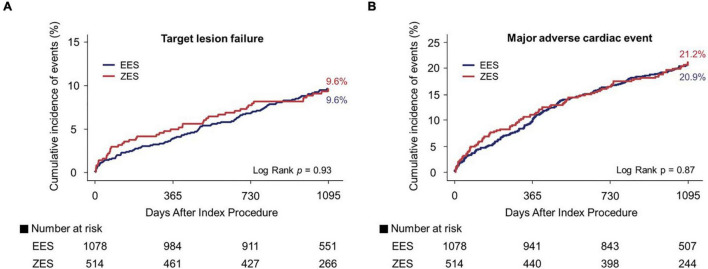
Kaplan–Meier curves for 3-year target lesion failure (TLF) and major adverse cardiac events (MACE). TLF **(A)** and MACE **(B)**. EES, everolimus-eluting stent; ZES, zotarolimus-eluting stent.

**FIGURE 4:**
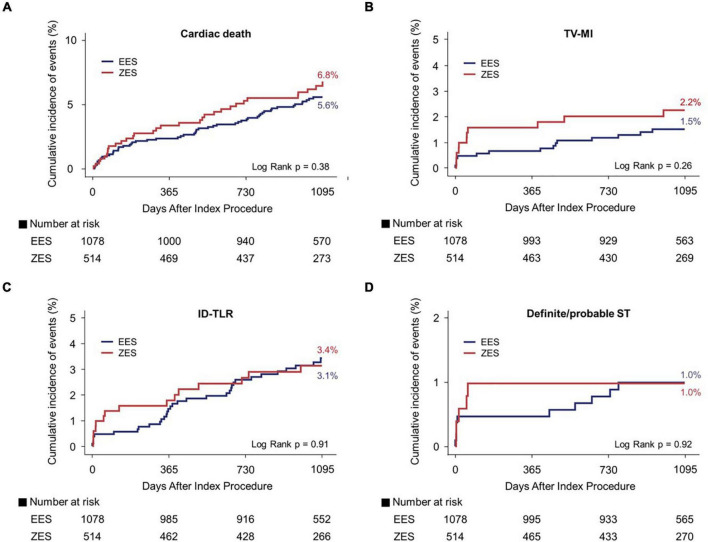
Kaplan–Meier curves for 3-year cardiac death, TV-MI, ID-TLR, and definite/probable ST. Cardiac death **(A)**, TV-MI **(B)**, ID-TLR **(C)**, and definite/probable ST **(D)**. TV-MI, target vessel myocardial infarction; ID-TLR, ischemic-driven target lesion revascularization; ST, stent thrombosis; EES, everolimus-eluting stent; ZES, zotarolimus-eluting stent.

Figures 3, 4 and Table 3 present a comparison of 3-year clinical outcomes between the two groups. Follow-up to 3 years was completed in 96.0% of all patients with a median follow-up duration of 1,095 days (interquartile range: 1,051–1,095 days). At 3 years, the risk of TLF was not significantly different between the two groups (9.6% in the EES group vs. 9.6% in the ZES group; hazard ratio [HR]: 0.98; 95% confidence interval [CI]: 0.69–1.40; *p* = 0.93). The MACE rates were also not significantly different between the two groups (EES vs. ZES, 20.9 vs. 21.2%; HR: 0.98; 95% CI: 0.78–1.24; *p* = 0.87). In addition, the individual components of TLF and MACE and definite/probable ST were also not different between the two groups. Consistent results were found in sensitivity analyses, such as multivariate Cox regression and IPTW analyses. Landmark analyses for the TLF and MACE were conducted in the overall population, setting the landmark points at 1 year (Supplementary Figure 1). Landmark analyses showed no difference in the incidence of the TLF and MACE between two groups within 1 year and between 1 and 3 years.

**TABLE 3:** Three-year outcome.

	EES (*n* = 1,078)	ZES (*n* = 514)	Unadjusted		Multivariable-adjusted		IPTW-adjusted	
					
			HR (95% CI)	*p*-value	HR (95% CI)	*p*-value	HR (95% CI)	*p*-value
**Primary outcome**								
TLF*	96 (9.6)	46 (9.6)	0.98 (0.69–1.40)	0.93	0.97 (0.67–1.40)	0.87	0.94 (0.67–1.33)	0.74
**Secondary outcome**								
MACE†	214 (20.9)	103 (21.2)	0.98 (0.78–1.24)	0.87	0.99 (0.77–1.26)	0.92	0.95 (0.76–1.20)	0.68
All death	96 (9.5)	53 (11.0)	0.85 (0.61–1.19)	0.35	0.86 (0.61–1.23)	0.41	0.86 (0.61–1.20)	0.36
Cardiac death	56 (5.6)	32 (6.8)	0.83 (0.53–1.27)	0.39	0.84 (0.53–1.34)	0.47	0.82 (0.53–1.26)	0.36
All MI	39 (3.9)	21 (4.6)	0.88 (0.52–1.49)	0.62	0.93 (0.54–1.61)	0.80	0.85 (0.51–1.44)	0.55
TV-MI	15 (1.5)	11 (2.2)	0.64 (0.30–1.40)	0.26	0.58 (0.26–1.28)	0.18	0.60 (0.28–1.28)	0.19
Any revascularization	113 (11.5)	53 (11.2)	1.00 (0.72–1.39)	0.99	1.01 (0.72–1.42)	0.94	0.95 (0.69–1.30)	0.74
ID-TLR	33 (3.4)	15 (3.1)	1.04 (0.56–1.91)	0.91	0.96 (0.52–1.79)	0.90	0.93 (0.52–1.67)	0.82
Definite/probable ST	10 (1.0)	5 (1.0)	0.95 (0.32–2.76)	0.92	0.82 (0.27–2.47)	0.72	0.80 (0.29–2.21)	0.67

*ZES, zotarolimus-eluting stent; EES, everolimus-eluting stent; ZES, zotarolimus-eluting stent; HR, hazard ratio; TLF, target lesion failure; MACE, major adverse cardiac events; TV-MI, target vessel myocardial infarction; ID-TLR, ischemic-driven target lesion revascularization; ST, stent thrombosis; IPTW, inverse probability of treatment weighting.*

*Data are presented as the number and percentages, along with Kaplan–Meier estimates.*

*Included (cardiac death, TV-MI, or ID-TLR).

*† Included (all death, all MI, or all revascularization).*

### Subgroup Analysis

**FIGURE 5:**
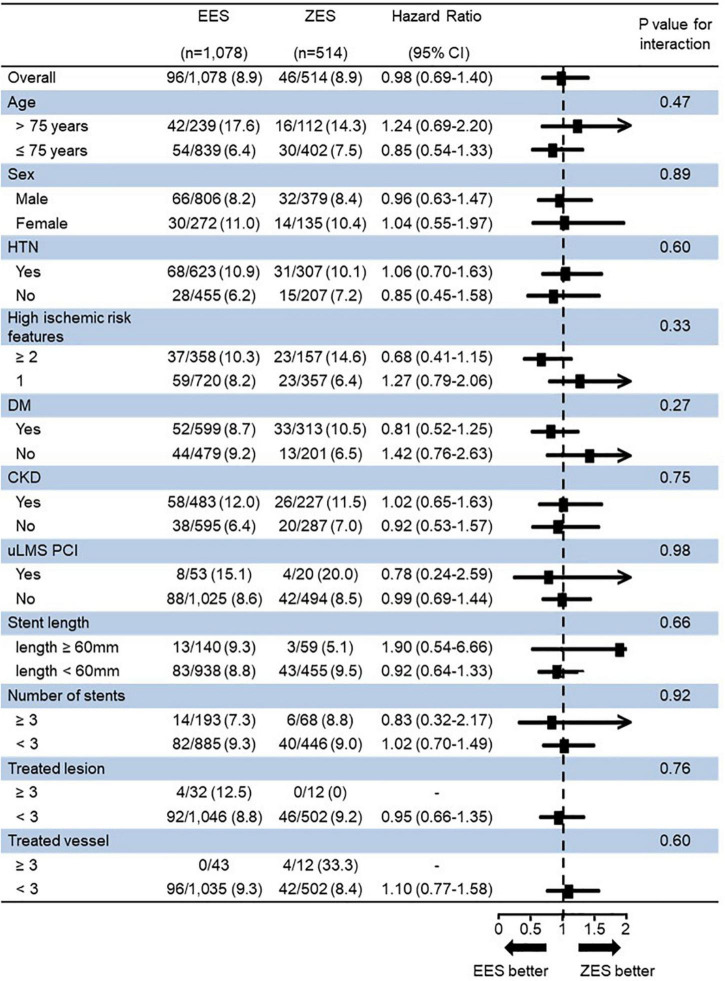
Subgroup analysis for 3-year target lesion failure (TLF). TLF, target lesion failure; EES, everolimus-eluting stent; ZES, zotarolimus-eluting stent; HTN, hypertension; DM, diabetes mellitus; CKD, chronic kidney disease; uLMS PCI, unprotected left main stem percutaneous coronary intervention.

Figure 5 presents a forest plot showing DES’s prognostic impact on the TLF among the various subgroups. In an exploratory subgroup analysis, the similar risk of TLF observed in the EES vs. ZES group was consistent across all subgroups; moreover, there was no significant interaction among the subgroups.

### Independent Predictors of Target Lesion Failure in Stent Thrombosis-Elevation Myocardial Infarction Patients With a High Ischemic Risk

A multivariable Cox proportional hazards model revealed independent predictors of 3 year TLF in STEMI patients with a high risk of ischemic events. Elderly patients (age > 75 years) (HR: 5.28, 95% CI: 1.90–14.66, *p* < 0.01) and uLMS PCI (HR: 7.68, 95% CI: 2.46–23.94, *p* < 0.01) were identified as 3-year TLF’s independent predictors, respectively. The MACE independent predictors at 3 years were elderly patients (age > 75 years), hypertension, prior cerebrovascular accident history, CKD, and Killip class ≥3 (Table 4 and Supplementary Table 5).

**TABLE 4:** Independent predictors for target lesion failure at 3 years.

Variables	Univariate analysis		Multivariate analysis^†^	
		
	HR (95% CI)	*P*-value	HR (95% CI)	*P*-value
Everolimus-eluting stent	0.98 (0.69–1.40)	0.93		
Age > 75 years	2.71 (1.94–3.78)	<0.01	5.28 (1.90–14.66)	<0.01
Body mass index, kg/m^2^	0.92 (0.87–0.98)	<0.01	1.03 (0.88–1.21)	0.69
Male gender	0.74 (0.52–1.06)	0.097	0.60 (0.22–1.67)	0.33
Hypertension	1.72 (1.20–2.45)	<0.01	0.54 (0.21–1.38)	0.20
Prior MI	1.67 (0.98–2.85)	0.06	1.09 (0.24–4.85)	0.91
Prior CVA	2.31 (1.44–3.70)	<0.01	2.12 (0.63–7.16)	0.23
CKD (eGFR < 60)	1.89 (1.35–2.65)	<0.01	1.53 (0.60–3.92)	0.38
Killip class 3/4	0.46 (0.32–0.65)	<0.01	1.38 (0.52–3.72)	0.53
LVEF < 40%	2.23 (0.97–5.09)	0.06	1.56 (0.59–4.14)	0.37
Cardiogenic shock	0.96 (0.56–1.64)	0.88		
uLMS PCI	1.97 (1.09–3.56)	0.03	7.68 (2.46–23.94)	<0.01

*HR, hazard ratio; CI, confidence interval; TLF, target lesion failure; MI, myocardial infarction; CVA, cerebrovascular accident; CKD, chronic kidney disease; eGFR, estimated glomerular filtration rate; LVEF, left ventricular ejection fraction; uLMS PCI, unprotected left main stem percutaneous coronary intervention.*

*†Any variable with p < 0.10 on univariate analysis was included in the multivariate models.*

## Discussion

The current study had the following principal findings: (1) clinical or lesion- or procedure-related high ischemic risk feature prevalence was observed in 43.4% of STEMI patients; (2) there were comparable 3-year clinical outcomes between the EES and ZES groups (Graphical Abstract); and (3) elderly patients (age > 75 years) and uLMS PCI were identified as 3-year TLF’s independent predictors in STEMI patients with a high risk of ischemic events.

In the present study, we found that approximately two-fifths (43.4%) of patients with STEMI had at least one high ischemic risk feature, such as DM, CKD, or complex procedures history (Supplementary Table 6). The current study excluded chronic total occlusion or bifurcation PCI with a two-stenting technique, which was included as a high ischemic risk from the previous criteria because those situations were relatively rare in STEMI when compared with stable ischemic heart disease and non-ST-elevation acute coronary syndrome, and these factors could not be identified in this registry data (5, 8, 9, 13). Worse clinical outcome predictors in the setting of STEMI and high ischemic risk have not yet been investigated, and the current study showed that elderly patients (age > 75 years) and uLMS PCI were 3-year TLF’s independent predictors. The elderly patients often have more multi-vessel disease and more complex coronary anatomy that include tortuosity and severely calcified lesion (14, 15), leading to higher adverse clinical outcomes after PCI. Therefore, PCI for elderly patients with AMI is often more technically challenging, such as advancing balloons and stenting to the lesion, and it could be difficult to achieve stent optimization. Cutting balloon angioplasty and rotational atherectomy are used to overcome the severely calcified lesion, and a better prognosis can be expected through these procedures for calcified plaque modification, but it is not easy to perform such a complex procedure for culprit lesion in the setting of STEMI (16). Regarding elderly patients as the predictors of TLF in STEMI patients with high ischemic risk, it might be explained that the ischemic adverse events increase due to the limitation of stent optimization by lesion complexity in the elderly patients. Therefore, in the STEMI with high ischemic features, it is necessary to judge the ischemic risk and bleeding risk well and decide an antiplatelet strategy, such as the usage of potent P2Y_12_ inhibitor or dual-antiplatelet therapy (DAPT) duration, carefully consideration of the vulnerability to the bleeding event, especially in elderly patients. Further dedicated research is needed to establish the high-risk factor for worse clinical outcomes in AMI patients with high ischemic features.

There are several studies from the registry data with heterogeneous designs and results reported that during the 3-year follow-up period, EES implantation led to better clinical outcomes, which was mainly driven by TLR when compared with ZES implantation (17). On the other hand, Mario et al. reported that clinical outcomes were similar for 5 years among patients with STEMI treated with ZES and EES (18). More recently, Kim et al. reported that similar 6-month clinical outcomes were observed between EES and ZES in patients with AMI (19). Although the previous two studies were conducted in patients with STEMI, they used early generation DESs and studied with a limited number of patients. The last study used new-generation DESs; however, it was not a dedicated study that analyzed only patients with STEMI, and only short-term clinical outcomes of 6 months were reported. Compared to previous studies, the current study evaluated relatively long-term clinical outcomes between EES and ZES groups.

From the current study, the eluted drug differences between EES and ZES did not show clinical outcome differences even in STEMI patients with high ischemic risks. This could be attributed to the following: second-generation permanent polymer DES has improved metallic design to further enhance the flexibility and deliverability in the complex lesion as compared to the first-generation DES; the polymers in the EESs and ZESs showed enhanced biocompatibility (20); and strut thickness in the EESs and ZESs was also similar, and both stents have sufficiently thin strut thickness (81–91 μm). Similar arterial healing properties are related to lower arterial drug concentrations observed in preclinical models, which may exert a favorable effect on endothelial maturation around stent struts (21). Moreover, optical coherence tomography demonstrated no significant differences between ZES and EES for tissue coverage, malapposition, and neointimal thickness during follow-up periods (22, 23). Based on these findings, EES and ZES may have similar short- and long-term efficacy and safety outcomes. It is expected that our study results could help the interventional cardiologists to choose DES types when considering primary PCI in extremely high ischemic risk situations.

The current study has several limitations. This study was a non-randomized, observational study, which has inherent selection and information biases. However, sensitivity analyses with IPTW analysis were conducted to adjust for the measured or unmeasured confounding factors. Second, patients who did not complete follow-up by 3 years were 40 (3.7%) and 23 (4.5%) in the EES and ZES groups, respectively. Third, there were no detailed procedural data, such as whether post-dilation has been performed or on the maximum balloon pressure and total procedure time. Fourth, the trends in the drug usage, such as antiplatelet agents, during the 3-year follow-up period were investigated, but the detailed reason for the change of the drug regimen was not determined. Furthermore, we did not consider the clinical impact of the various DAPT regimens, such as escalation and de-escalation (Supplementary Table 7). Fifth, except for the eluted drug, the effect of the difference in the stent design, polymer, and thickness was not considered.

## Conclusion

Primary PCI with EES or ZES provided comparable clinical outcomes, including 1.0% of definite/probable ST at 3 years, in STEMI patients with high ischemic risks. These findings were confirmed based on the dedicated AMI registry, demonstrating the efficacy and safety of contemporary second-generation DES for the treatment of STEMI patients with a risk of ischemic events.

## Key Messages

(1) In clinical practice, the prevalence of clinical, lesion-, or procedure-related high ischemic risk features was observed in 43.4% of the patients with STEMI.
(2) Primary percutaneous coronary intervention (PCI) with everolimus-eluting stent (EES) or zotarolimus-eluting stent (ZES) provided comparable clinical outcomes in STEMI patients with a high ischemic risk.
(3) Elderly patients (age > 75 years) and unprotected left main stem (uLMS) PCI were identified as independent predictors of 3-year target lesion failure (TLF) in STEMI patients with a high risk of ischemic events.

## Data Availability Statement

The raw data supporting the conclusions of this article will be made available by the authors, without undue reservation.

## Ethics Statement

The studies involving human participants were reviewed and approved by the Institutional Review Board of Chonnam National University Hospital. The patients/participants provided their written informed consent to participate in this study.

## Author Contributions

O-HL and YK contributed to the study concept and design. YK, D-KC, J-SK, B-KK, DC, M-KH, MJ, and YJ acquired the data. D-KC, DC, M-KH, and YJ supervised the progress of the study. O-HL, YK, and MJ contributed to the acquisition, analysis, and interpretation of data, drafting for the manuscript, and critical revision of the manuscript for important intellectual content. All authors listed have made a substantial, direct, and intellectual contribution to the work, and approved it for publication.

## Conflict of Interest

The authors declare that the research was conducted in the absence of any commercial or financial relationships that could be construed as a potential conflict of interest.

## Publisher’s Note

All claims expressed in this article are solely those of the authors and do not necessarily represent those of their affiliated organizations, or those of the publisher, the editors and the reviewers. Any product that may be evaluated in this article, or claim that may be made by its manufacturer, is not guaranteed or endorsed by the publisher.

## Abbreviations

DP: durable polymers
EES: everolimus-eluting stent
ID-TLR: ischemia-driven target lesion revascularization
KAMIR-NIH: Korea Acute Myocardial Infarction Registry-National Institute of Health
MACE: major adverse cardiovascular event
ST: stent thrombosis
TLF: target lesion failure
TV-MI: target vessel myocardial infarction
ZES: zotarolimus-eluting stent.
