# Corrigendum: Safety and efficacy of contemporary drug-eluting stents in patients with ST-segment elevation myocardial infarction and a high ischemic risk

**DOI:** 10.3389/fcvm.2022.952331

**Published:** 2022-08-24

**Authors:** Oh-Hyun Lee, Yongcheol Kim, Nak-Hoon Son, Deok-Kyu Cho, Jung-Sun Kim, Byeong-Keuk Kim, Donghoon Choi, Myeong-Ki Hong, Myung Ho Jeong, Yangsoo Jang

**Affiliations:** ^1^Yonsei University College of Medicine Cardiovascular Center, Yongin Severance Hospital, Yongin-si, South Korea; ^2^Data Science Team (Biostatistician), Center for Digital Health, Yongin Severance Hospital, Yongin-si, South Korea; ^3^Department of Statistics, Keimyung University, Daegu, South Korea; ^4^Severance Cardiovascular Hospital, Yonsei University Health System, Seoul, South Korea; ^5^Chonnam National University Hospital and Medical School, Gwangju, South Korea; ^6^Department of Cardiology, CHA Bundang Medical Center, CHA University School of Medicine, Seongnam-si, South Korea

**Keywords:** ST-segment elevation myocardial infarction, drug-eluting stent, percutaneous coronary intervention (PCI), target lesion failure, high risk factor

In the published article, there was an error in affiliation 5. Instead of “Chonnam National University Hospital, Gwangju, South Korea,” it should be “Chonnam National University Hospital and Medical School, Gwangju, South Korea.”

The authors apologize for this error and state that this does not change the scientific conclusions of the article in any way. The original article has been updated.

## Publisher's note

All claims expressed in this article are solely those of the authors and do not necessarily represent those of their affiliated organizations, or those of the publisher, the editors and the reviewers. Any product that may be evaluated in this article, or claim that may be made by its manufacturer, is not guaranteed or endorsed by the publisher.

