# Supplementary material for: Safety and Efficacy of Contemporary Drug-Eluting Stents in Patients With ST-Segment Elevation Myocardial Infarction and a High Ischemic Risk
Source: Front Cardiovasc Med. 2022 May 23;9:880351. doi: 10.3389/fcvm.2022.880351 (PMC9167955; doi:10.3389/fcvm.2022.880351)

**Supplementary Material**

**Safety and Efficacy of Contemporary Drug-Eluting Stents in Patients with ST-Segment Elevation Myocardial Infarction and a High Ischemic Risk**

Oh-Hyun Lee, MD^1†^, Yongcheol Kim, MD, PhD^1†^, Nak-Hoon Son, PhD^2,3^, Deok-Kyu Cho, MD^1^, Jung-Sun Kim, MD, PhD^4^, Byeong-Keuk Kim, MD, PhD^4^, Donghoon Choi, MD, PhD^1^, Myeong-Ki Hong, MD, PhD^4^, Myung Ho Jeong, MD, PhD^5*^, Yangsoo Jang, MD, PhD^6^; on behalf of the KAMIR-NIH Investigators

**CONTENTS**

**Supplementary Table 1. Characteristics of all second-generation DES utilized in the study**...........................................................................................................................................1

**Supplementary Table 2. Propensity score information**….......….……..…….…….………3

**Supplementary Table 3. Baseline characteristics in the IPTW-adjusted cohort……………………………**…………………………………...………….…….………5

**Supplementary Table 4. Angiographic and procedural characteristics in the IPTW-adjusted cohort** ……………………………………………………...……………….………7

**Supplementary Table 5. Independent predictors for MACE at three years**………………………………………………………………………………..………….9

**Supplementary Table 6. Proportions of a high ischemic risk in the study population**…………………………………………………………………………………...10

**Supplementary Table 7. Medication use**…………………………......….………….……..11

**Supplementary Figure 1. Landmark analysis of TLF and MACE**....................................12

**Supplemental Tables**

**Supplementary Table 1. Characteristics of all second-generation DES utilized in the study**

|  | Everolimus-eluting stent | | Zotarolimus-eluting stent | |
| --- | --- | --- | --- | --- |
|  | Xience (Prime, Expedition, Alpine) | Promus (Element, Premier) | Resolute Integrity | Resolute Onyx |
| Strut cross section |  |  |  |  |
| Drug | Everolimus | Everolimus | Zotarolimus | Zotarolimus |
| Dose, ug/mm^2^ | 1.0 | 1.0 | 1.6 | 1.6 |
| Polymer | PBMA/PVDF-HFP | PVDF-HFP | BioLinx | BioLinx |
| Type | Durable | Durable | Durable | Durable |
| Coating | Circumferential | Circumferential | Circumferential | Circumferential |
| Thickness, μm | 7-8 | 7-8 | 6 | 6 |
| Load, μg | 232 | 241.8 | 380 | 317 |
| Strut thickness, μm | 81 | 81  86 (only 4.0mm stent) | 91 | 81 |
| Strut width, μm | 100 | 67  89 (only 4.0mm stent) | 91 | 91 |
| Metal alloy | Co-Cr | Pt-Cr | Co-Ni | Co-Cr: shell  Pt-Ir: core |

PBMA indicates poly n-butyl methacrylate; PVDF-HFP, polyvinylidenefluoropropylene; Co-Cr, cobalt-chromium; Pt-Cr, platinum-chromium; Co-Ni, cobalt-nickel; Pt-Ir, platinum-iridium.

**Supplementary Table 2. Propensity score information**

| Propensity Score Information | | | | | | | | | | | | | | | | | | | | | | |
| --- | --- | --- | --- | --- | --- | --- | --- | --- | --- | --- | --- | --- | --- | --- | --- | --- | --- | --- | --- | --- | --- | --- |
| Observations | Treated (EES) | | | | | | | | | Control (ZES) | | | | | | | | | Treated - Control | | | |
|  | N | Weight | | Mean | Standard Deviation | | Min | | Max | N | Weight | | Mean | Standard Deviation | | Min | Max | | Mean Difference | | | |
| All | 1078 |  | | 0.6811 | 0.0522 | | 0.5582 | | 0.8113 | 514 |  | | 0.6687 | 0.0512 | | 0.5582 | 0.8113 | | 0.0124 | | | |
| Region | 1078 |  | | 0.6811 | 0.0522 | | 0.5582 | | 0.8113 | 514 |  | | 0.6687 | 0.0512 | | 0.5582 | 0.8113 | | 0.0124 | | | |
| Weighted | 1053 | 1592.01 | | 0.6771 | 0.0521 | | 0.5582 | | 0.8113 | 509 | 1591.36 | | 0.6670 | 0.0517 | | 0.5582 | 0.8113 | | 0.001 | | | |
| Standardized Mean Differences (Treated – Control) | | | | | | | | | | | | | | | | | | | | | |  |
| Variable | | | Observations | | | Mean Difference | | Standard Deviation | | | | Standardized Difference | | | Percent Reduction | | | Variance Ratio | | |  |  |
| Total Stent Number | | | All | | | 0.12417 | | 0.86817 | | | | 0.14303 | | |  | | | 1.1997 | |  |  |  |
|  | | | Region | | | 0.12417 | |  | | | | 0.14303 | | | 0 | | | 1.1997 | |  |  |  |
|  | | | Weighted | | | 0.0036 | |  | | | | 0.00414 | | | 97.1 | | | 0.9897 | |  |  |  |
| DM | | | All | | | 0.05329 | | 0.492459 | | | | 0.10821 | | |  | | | 1.0368 | |  |  |  |
|  | | | Region | | | 0.05329 | |  | | | | 0.10821 | | | 0 | | | 1.0368 | |  |  |  |
|  | | | Weighted | | | 0.00183 | |  | | | | 0.00371 | | | 96.57 | | | 1.0011 | |  |  |  |
| Clopidogrel | | | All | | | 0.05126 | | 0.448508 | | | | 0.11429 | | |  | | | 1.1185 | |  |  |  |
|  | | | Region | | | 0.05126 | |  | | | | 0.11429 | | | 0 | | | 1.1185 | |  |  |  |
|  | | | Weighted | | | 0.00175 | |  | | | | 0.0039 | | | 96.59 | | | 1.0036 | |  |  |  |
| Multivessel Treated | | | All | | | -0.05811 | | 0.446141 | | | | -0.13026 | | |  | | | 1.1399 | |  |  |  |
|  | | | Region | | | -0.05811 | |  | | | | -0.13026 | | | 0 | | | 1.1399 | |  |  |  |
|  | | | Weighted | | | -0.00256 | |  | | | | -0.00574 | | | 95.6 | | | 1.0054 | |  |  |  |
| Imaging | | | All | | | 0.04348 | | 0.410674 | | | | 0.10587 | | |  | | | 0.8634 | |  |  |  |
|  | | | Region | | | 0.04348 | |  | | | | 0.10587 | | | 0 | | | 0.8634 | |  |  |  |
|  | | | Weighted | | | -0.0014 | |  | | | | -0.0034 | | | 96.79 | | | 1.005 | |  |  |  |
| Standard deviation of All observations used to compute standardized differences | | | | | | | | | | | | | | | | | | | | | |  |

**Supplementary Table 3. Baseline characteristics in the IPTW-adjusted cohort**

|  | IPTW-adjusted cohort | | |
| --- | --- | --- | --- |
|  | EES (n=1,053) | ZES (n=509) | *p* value |
| Age, y | 64.9 ± 11.7 | 65.2 ± 12.0 | 0.70 |
| Male gender, n (%) | 806 (74.8) | 387 (73.7) | 0.65 |
| Body mass index,† kg/m^2^ | 24.0 ± 3.2 | 24.2 ± 3.1 | 0.28 |
| Hypertension, n (%) | 623 (57.8) | 312 (59.4) | 0.53 |
| Diabetes mellitus, n (%) | 599 (55.6) | 317 (60.4) | 0.07 |
| Dyslipidemia, n (%) | 131 (12.2) | 73 (13.9) | 0.32 |
| Current smoker, n (%) | 412 (38.2) | 198 (37.7) | 0.85 |
| Prior myocardial infarction, n (%) | 74 (6.9) | 36 (6.9) | 0.99 |
| Prior cerebrovascular accident, n (%) | 75 (7.0) | 38 (7.2) | 0.84 |
| Killip class ≥ 3, n (%) | 198 (18.4) | 91 (17.3) | 0.61 |
| LVEF ≤ 40%, n (%) | 51 (24.2) | 17 (17.5) | 0.24 |
| Cardiogenic shock | 117 (10.9) | 60 (11.4) | 0.73 |
| Laboratory findings |  |  |  |
| Peak troponin I, pg/ml | 79.7 ± 104.6 | 71.8 ± 108.7 | 0.20 |
| LDL-cholesterol, mg/dL | 109.3 ± 38.7 | 109.9 ± 38.1 | 0.79 |
| Creatinine, mg/dL | 1.2 ± 0.8 | 1.2 ± 0.9 | 0.87 |
| Hemoglobin, g/dL | 13.8 ± 2.0 | 13.9 ± 2.2 | 0.80 |
| Platelet count, 10^3^/μL | 231.2 ± 64.5 | 237.2 ± 69.6 | 0.10 |
| Discharge medication, n (%) |  |  |  |
| Aspirin | 1069 (99.2) | 524 (99.8) | 0.18 |
| P2Y_12_ inhibitor |  |  |  |
| Clopidogrel | 748 (69.4) | 384 (73.1) | 0.12 |
| Prasugrel | 100 (9.3) | 43 (8.2) | 0.47 |
| Ticagrelor | 225 (29.1) | 94 (27.8) | 0.67 |
| ACEi or ARB | 845 (78.4) | 407 (77.5) | 0.70 |
| Beta-blocker | 949 (88.0) | 458 (87.2) | 0.65 |
| Calcium channel blocker | 42 (3.9) | 24 (4.6) | 0.52 |
| Statin | 1014 (94.1) | 486 (92.6) | 0.25 |

IPTW indicates inverse probability of treatment weighting; ZES, zotarolimus-eluting stent; EES, everolimus-eluting stent; LVEF, left ventricular ejection fraction; LDL, low density lipoprotein; CRP, C-reactive protein, ACEi, angiotensin converting enzyme inhibitor; ARB, angiotensin receptor blocker.

*Plus-minus values are mean ± standard deviation.

†The body mass index is the weight in kilograms divided by the square of the height in meters.

**Supplementary Table 4. Angiographic and procedural characteristics in the IPTW-adjusted cohort**

|  | IPTW-adjusted cohort | | | | | |  |
| --- | --- | --- | --- | --- | --- | --- | --- |
|  | EES (n=1,053) | | ZES (n=509) | | *p* value | |  |
| Trans-radial approach, n (%) | | 225 (20.9) | | 114 (21.7) | | 0.70 | |
| Target vessel, n (%) | |  | |  | | 0.24 | |
| LM | | 31 (2.9) | | 15 (2.9) | |  | |
| LAD | | 527 (48.9) | | 241 (45.9) | |  |  |
| LCX | | 81 (7.5) | | 30 (5.7) | |  |  |
| RCA | | 439 (40.7) | | 239 (45.5) | |  |  |
| CAD extent, n (%) | |  | |  | | 0.45 | |
| CAD 1VD | | 430 (39.9) | | 221 (42.1) | |  | |
| CAD 2VD | | 397 (36.8) | | 196 (37.3) | |  |  |
| CAD 3VD | | 251 (23.3) | | 108 (20.6) | |  |  |
| LM involvement, n (%) | | 69 (6.4) | | 25 (4.8) | | 0.19 | |
| Lesion type B2 or C, n (%) | | 972 (90.2) | | 461 (87.8) | | 0.15 | |
| Multiple treated vessels (≥ 2), n (%) | | 329 (30.5) | | 138 (26.3) | | 0.08 | |
| Multiple stents (≥ 2), n (%) | | 365 (43.1) | | 203 (38.7) | | 0.09 | |
| Mean stent diameter, mm | | 3.1 ± 0.4 | | 3.2 ± 0.4 | | 0.20 | |
| Total stent number | | 1.7 ± 0.9 | | 1.6 ± 0.9 | | 0.09 | |
| Total stent length, mm | | 34.6 ± 16.8 | | 33.6 ± 16.5 | | 0.25 | |
| Imaging-guided PCI, n (%) | | 209 (19.4) | | 122 (23.2) | | 0.07 | |
| Thrombus aspiration, n (%) | | 351 (32.7) | | 182 (35.2) | | 0.32 | |
| Glycoprotein IIb/IIIa inhibitor use, n (%) | | 234 (21.7) | | 99 (18.9) | | 0.19 | |

IPTW indicates inverse probability of treatment weighting; ZES, zotarolimus-eluting stent; EES, everolimus-eluting stent; LM, left main artery, LAD, left anterior descending artery; LCX, left circumflex artery; RCA, right coronary artery; CAD, coronary artery disease; LM, left main; PCI, percutaneous coronary intervention.

*Plus-minus values are mean ± standard deviation.

**Supplementary Table 5. Independent predictors for MACE at three years**

| Variables | Univariate Analysis | | Multivariate Analysis^†^ | | |  |
| --- | --- | --- | --- | --- | --- | --- |
|  | HR (95% CI) | P value | | HR (95% CI) | P value | |
| Everolimus-eluting stent | 0.98 (0.78-1.24) | 0.87 | |  |  | |
| Age > 75 years | 1.73 (1.36-2.20) | <0.01 | | 1.41 (1.09-1.83) | <0.01 | |
| Hypertension | 1.47 (1.16-1.85) | <0.01 | | 1.36 (1.07-1.72) | 0.01 | |
| Current smoker | 0.81 (0.64-1.02) | 0.07 | | 0.97 (0.76-1.24) | 0.83 | |
| Prior CVA | 1.88 (1.34-2.66) | <0.01 | | 1.56 (1.10-2.21) | 0.01 | |
| CKD (eGFR<60) | 1.55 (1.24-1.93) | <0.01 | | 1.28 (1.02-1.62) | 0.04 | |
| Killip class 3/4 | 1.73 (1.34-2.22) | <0.01 | | 1.58 (1.22-2.04) | <0.01 | |

MACE indicates major adverse cardiac event; HR, hazard ratio; CVA, cerebrovascular accident; CKD, chronic kidney disease; eGFR, estimated glomerular filtration rate.

†Any variable with P < 0.10 on univariate analysis was included in the multivariate models.

|  | 6,325 STEMI patients in the KAMIR-NIH registry | | |
| --- | --- | --- | --- |
|  | STEMI with a high ischemic risk (n = 2,744) | EES (n = 1,078) | ZES  (n = 514) |
| High ischemic features ≥ 2 | 870 (31.7) | 358 (33.2) | 157 (30.5) |
| DM | 1583 (57.7) | 599 (55.6) | 313 (60.9) |
| CKD | 1317 (48.0) | 483 (44.8) | 227 (44.2) |
| uLMS PCI | 136 (5.0) | 53 (4.9) | 20 (3.9) |
| Stent length ≥ 60 mm | 242 (8.8) | 140 (13.0) | 59 (11.5) |
| Implanted stents ≥ 3 | 411 (15.0) | 193 (17.9) | 68 (13.2) |
| Treated lesion ≥ 3 | 61 (2.2) | 32 (3.0) | 12 (2.3) |
| Three-vessels treated | 86 (3.1) | 43 (4.0) | 12 (2.3) |

**Supplementary Table 6. Proportions of a high ischemic risk in study population**

STEMI indicates ST-elevation myocardial infarction; EES, everolimus-eluting stent; ZES, zotarolimus-eluting stent; DM, diabetes mellitus; CKD, chronic kidney disease; uLMS PCI, unprotected left main stem percutaneous coronary intervention.

**Supplementary Table 7. Medication use**

|  | At discharge | | | At 1 year | | | At 2 years | | | At 3 years | | |
| --- | --- | --- | --- | --- | --- | --- | --- | --- | --- | --- | --- | --- |
|  | EES | ZES | P value | EES | ZES | P value | EES | ZES | P value | EES | ZES | P value |
|  | (n=1,078) | (n=514) |  | (n=994) | (n=476) |  | (n=929) | (n=435) |  | (n=870) | (n=403) |  |
| Antiplatelet agent |  |  |  |  |  |  |  |  |  |  |  |  |
| Aspirin | 1069 (99.2) | 513 (99.8) | 0.18 | 903 (90.8) | 442 (92.9) | 0.20 | 748 (80.5) | 364 (83.7) | 0.16 | 648 (74.5) | 316 (78.4) | 0.13 |
| P2Y_12_ inhibitor | 1073 (99.5) | 510 (99.2) | 0.74 | 822 (82.7) | 364 (76.5) | 0.02 | 665 (71.6) | 261 (60.0) | <0.01 | 593 (68.2) | 242 (60.0) | <0.01 |
| Clopidogrel | 748 (69.7) | 383 (75.1) | 0.04 | 719 (87.5) | 321 (88.2) | 0.05 | 620 (93.2) | 246 (94.3) | <0.01 | 564 (95.1) | 230 (95.0) | <0.01 |
| Prasugrel | 100 (9.3) | 39 (7.6) | 0.26 | 41 (5.0) | 20 (5.5) | 1.00 | 22 (3.3) | 7 (2.7) | 0.43 | 14 (2.4) | 5 (2.1) | 0.81 |
| Ticagrelor | 225 (21.0) | 88 (17.3) | 0.43 | 62 (7.5) | 23 (6.3) | 0.34 | 23 (3.5) | 8 (3.1) | 0.56 | 15 (2.5) | 7 (2.9) | 1.00 |
| DAPT | 1066 (98.9) | 521 (99.2) | 0.60 | 752 (69.8) | 343 (65.3) | 0.07 | 512 (47.5) | 204 (38.9) | <0.01 | 399 (37.0) | 168 (32.0) | 0.049 |
| Anticoagulant agent | 39 (3.6) | 21 (4.1) | 0.67 | 34 (3.4) | 13 (2.7) | 0.53 | 34 (3.7) | 13 (3.0) | 0.63 | 35 (4.0) | 15 (3.7) | 0.88 |
| Triple Therapy | 38 (3.5) | 21 (4.1) | 0.57 | 11 (1.1) | 3 (0.6) | 0.57 | 7 (0.8) | 0 | 0.11 | 4 (0.5) | 1 (0.2) | 1.00 |
| Dual therapy | 1 (0.1) | 0 | 1.00 | 21 (2.1) | 10 (2.1) | 1.00 | 20 (2.2) | 12 (2.8) | 0.57 | 24 (2.8) | 13 (3.2) | 0.72 |
| Beta-blocker | 949 (88.0) | 448 (87.2) | 0.62 | 808 (81.3) | 392 (82.4) | 0.62 | 717 (77.2) | 352 (80.9) | 0.12 | 661 (76.0) | 322 (79.9) | 0.12 |
| RAS inhibitor | 845 (78.4) | 397 (77.2) | 0.61 | 733 (73.7) | 362 (76.1) | 0.34 | 660 (71.0) | 324 (74.5) | 0.19 | 625 (71.8) | 297 (73.7) | 0.49 |
| Statin | 1014 (94.1) | 476 (92.6) | 0.27 | 918 (92.4) | 444 (93.3) | 0.53 | 849 (91.4) | 403 (92.6) | 0.43 | 806 (92.6) | 381 (94.5) | 0.21 |

Data are presented as the number and percentage.

ZES indicates zotarolimus-eluting stent; EES, everolimus-eluting stent; DAPT, dual antiplatelet therapy; RAS, renin-angiotensin system.

**Supplementary Figure 1. Landmark analysis of TLF and MACE**


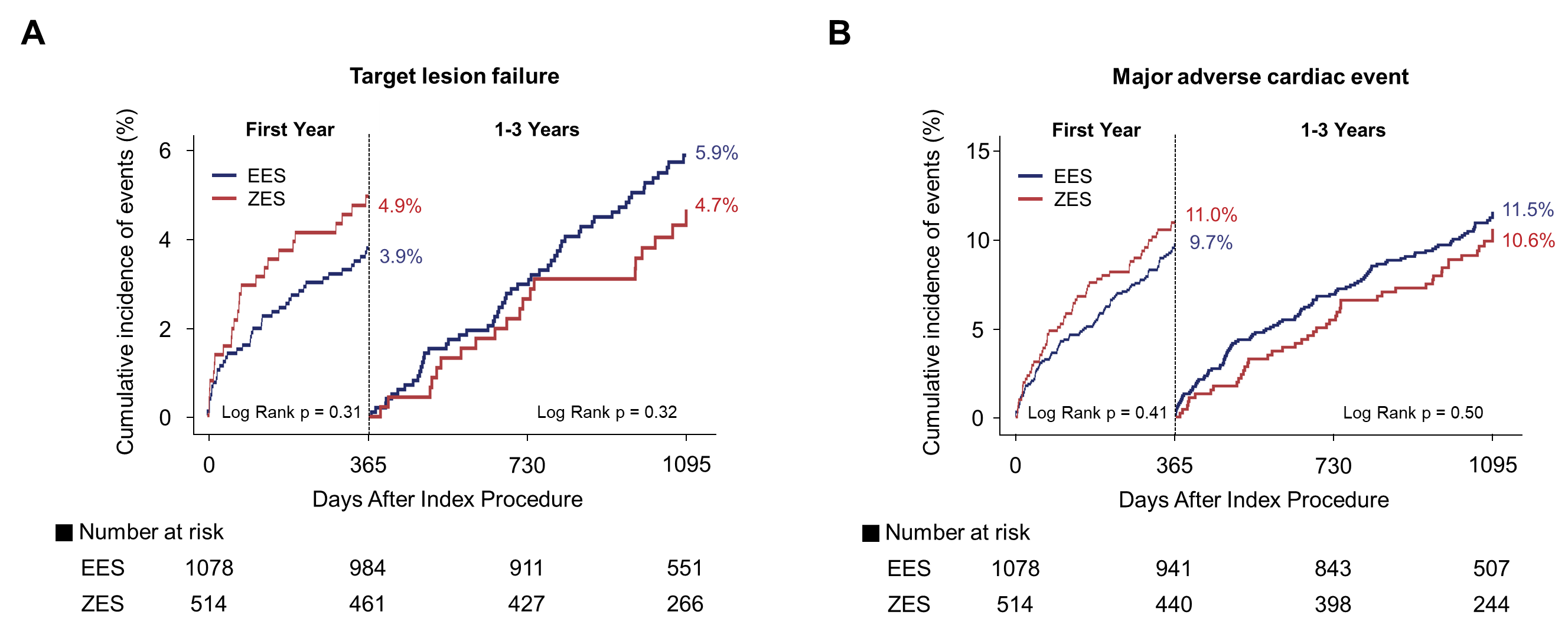

Supplement: Supplementary file 1 [file Data_Sheet_1.docx]
